# Supplementary material for: Optimal utilization of prevention of mother-to-child transmission of HIV services among adolescents under group versus focused antenatal care in Eastern Uganda
Source: PLoS One. 2022 Nov 1;17(11):e0275905. doi: 10.1371/journal.pone.0275905 (PMC9624396; doi:10.1371/journal.pone.0275905)
Supplement: S1 Table — (PDF) [file pone.0275905.s001.pdf]

Abbreviation	Complete Name
AIDS	Acquired Immune Deficiency Syndrome
ANC	Antenatal care
ARV	Antiretroviral therapy
DNA PCR	Deoxyribose Nucleic Acid Polymerase Chain Reaction
e-MTCT	Elimination of mother to child transmission (of HIV)
F-ANC	Focused Antenatal Care
G-ANC	Group Antenatal care
HCIV	Health Centre IV
HCT	HIV counseling and testing
HIV	Human immunodeficiency virus
MTCT	Mother to child transmission (of HIV)
NVP	Nevirapine
PMTCT	Prevention of Mother-to-Child Transmission (of HIV)
UNAIDS	Joint United Nations Program on HIV and AIDS
WHO	World Health Organization
