# Supplementary material for: Optimal utilization of prevention of mother-to-child transmission of HIV services among adolescents under group versus focused antenatal care in Eastern Uganda
Source: PLoS One. 2022 Nov 1;17(11):e0275905. doi: 10.1371/journal.pone.0275905 (PMC9624396; doi:10.1371/journal.pone.0275905)
Supplement: Attachment — Submitted filename: Response to reviewers.docx [file pone.0275905.s002.docx]

May 05, 2020

To: Orvalho Augusto

Academic Editor

PLOS ONE

Reference number: PONE-D-21-31253

Thank you for your consideration and for inviting us to submit the revised version of our manuscript entitled: “*Optimal utilization of Prevention of Mother-to-Child Transmission of HIV services among adolescents under Group versus Focused antenatal care in Eastern Uganda*.” The responses to each of the reviewers' comments are listed below.

**Responses to the reviewers’ comments**

**Academic editor**

**General comment**

This is quite an interesting report on a strategy to improve PMTCT among adolescents. As an alternative to individual antenatal care, the authors are testing a group antenatal care. As an outcome, the authors use something they called “optimal utilization of PMTCT of HIV services”. This is a compositive outcome with important components the authors decided not to look at.

**Response**

Thank you for this summary of the paper. Our responses to each of the individual comments are provided below.

According to the World Health Organization, the PMTCT strategy is a cascade of events that enable the identification of an HIV-infected pregnant woman and the prevention of HIV transmission to her infant. It comprises: attending antenatal care (ANC), HIV counseling and testing (HCT), and receiving HIV test results. If HIV negative, subsequent retesting in the third trimester, shortly after delivery, or within three months. If positive, antiretroviral therapy (ARVs) for the mother under option B plus (1), Nevirapine syrup (NVP), cotrimoxazole prophylaxis, and ARVs for the infant. Safe delivery, safe infant feeding, early infant diagnosis within 6 weeks, DNA PCR done 6 weeks after ceasing breastfeeding, serology at 18 months if DNA PCR is negative, and finally linkage into care and treatment if infant turns out positive (1) (2).

We therefore for HIV negative mothers considered first HIV counseling and testing before the third trimester, receipt of results and subsequent re-testing as recommended. For HIV positive mothers however, we looked at whether they were initiated on ART under option B plus. In addition, for the infants we asked if: there was safe infant delivery (delivery in a health facility), safe infant feeding (exclusive breast feeding for the first 6 months), infant testing, and receipt of results, nevirapine was given for six weeks, and cotrimoxazole was given until status was confirmed. Infant testing entailed early infant diagnosis (DNA PCR within 6 weeks), if the test turned positive, ART was initiated and the infant linked into care. We did not assess for the second DNA PCR test or serology as the infants had to be 9 months of age or less.

**Comment #1 (Grammar and spelling errors)**

There are so many avoidable English typos all over the manuscript. Please, do revise.

**Response**: This manuscript has been edited and the spelling errors are taken care of

**Comment #2 (Abstract)**

Please add in the abstract a short definition of this concept of “optimal utilization of PMTCT of HIV services”.

**Response:** This has been added on line 33 of the manuscript with track changes

**Comment #3 (Study design and setting)**

Line 85 in the study design: the rates presented there are yearly rates? Clarify these figures, please.

**Response**

The most current data on the adolescent pregnancy rate in the Eastern region is from 2016. This along with the HIV pregnancy rate in 2020 have been specified on line 111 in the study design and setting section.

**Comment #4 (Study participants and sampling)**

The eligibility criteria to be part of the study are quite unclear as the reviewer explains below. Did the women have to fulfill all the criteria in lines 99 to 101? Or just one criterion would be enough to participate in the study?

**Response**

All the criteria listed had to be met; that is, the mothers had to: a) have sought care during the study period, b) have had completed at least 18 months of care if HIV positive, and 9 months of care if HIV negative c) have alive infants 9 months or less and d) provided written informed consent. This has been clarified in line 127.

**Comment #5 (Sample size)**

Sample size: is the effect size an absolute difference of the proportion? Or a relative difference. Clarify.

**Response**

This was the absolute difference between the proportions and has been edited in line 136.

**Comment #6 (Variables)**

Line 124: How the exposure was measured here?

**Response**

At 2 health facilities (Jinja regional referral hospital and Budondo HCIV), F-ANC was the only mode of ANC available. However, at Iganga hospital and Bugembe HCIV, G-ANC was offered in addition to F-ANC. We checked the mode of ANC from their ANC card and/or the G-ANC register. We also asked the mothers whether they had received ANC care from any other health facilities and then assessed the ANC mode they had received. Line 155

**Comment #7 (Data management and statistical analysis)**

**-** Cite Epidata and Stata

**-** It is a bit problematic to use a mixed-effects model with just 4 clusters. Even with the sandwiched standard errors. I would suggest fitting fixed-effects models and keeping the health facility as 3 dummy indicators.

**-** Each one of the components of the “optimal utilization of PMTCT of HIV services” deserves to be analyzed. At least show the overall prevalence of these.

**-** Put the Ethical approval within the methods section

**-** There are so many abbreviations. Please provide a list

**Response**

- Epidata and stata have been cited lines 182-183
- The data have been re-analyzed using a fixed-effects model keeping the health facility as 3 dummy indicators. line 191.
- Each of the components of optimal utilization of PMTCT of HIV services has been analyzed using fixed effects regression modeling, and a prevalence ratio comparing G-ANC to F-ANC computed-line 191
- The ethical approval has been moved to the methods section- lines 202 to 205.
- A list of abbreviations has been provided as supporting information.

**Comment #8 (Results)**

Be consistent with the proportions. In the tables 1 and 2 they are presented as percentages. But on the text lines 171 to 173, for example, they are something else.

**Response**: These have been edited to reflect percentages in lines 224 to 225.

**Comment #9 (Table 1)**

Table 1:
- Add the age descriptives described on line 163.
- At the HIV test result. What are the 1 and 2 rows?
- the mean travel time how was measured? This is not described in the methods.
- Please add as footnote how these p-values were computed
For the analysis of optimal utilization in line 169 to 173 I would request to add here the different components of this composite measure. There is an interest to see at what component the intervention did make a change.

**Response**

- The age descriptives have been added in table 1 as recommended.
- Row 1 is HIV positive mothers while row 2 is HIV negative mothers. These have been reflected in table 1.
- The mean clinic travel time was measured by self-report whereby the mothers were asked how much time it took them to get to the clinic where they received ANC. This has been added in the “Variables” section of the methods on lines 160 to 162.
- The P- values were computed using the Pearson’s Chi-square test for categorical predictors and the Wilcoxon rank-sum test for continuous ones. This has been added as a footnote to table 1.
- Each component of the outcome has been analyzed for ANC mode. This has been stratified by HIV test results as shown in table 2 and fig1. Prevalence ratios for the HIV positive mothers have not been computed due to the small numbers in this group.

**Comment #10 (Table 2)**
- Add one column for prevalence of “optimal utilization of PMTCT services”
- For the first ANC, please change the reference category
- Age in the column of “Yes” we have 242. Add its proportion.
In fact, the analysis of factors here is not that important. It is OK to adjust for these factors to have something like table 3. But I would prefer to have the all composite elements analysed to get their PR for the ANC type.

**Response**

- A column for the prevalence of “optimal utilization of PMTCT services” has been added to table 3
- The reference category for first ANC has been changed to “first trimester of ANC” as shown in table 3
- The proportion for age category ≥18 years has been added in table 3
- Each component of the outcome has been analyzed for ANC type. This has been stratified by HIV test results as shown in table 2 and fig1.

**Comment #11 (Table 3)**

Table 3 - this is a multivariable analysis. Correct the lone 193 as well. How this model came? How these covariates were chosen?
- Change the reference for the first ANC

**Response**

-This has been edited to ‘multivariable analysis’ as advised.

-Prevalence ratios between individual predictors and the optimal utilization of PMTCT of HIV services at the bivariate level were assessed and those with a p-value less than or equal to 0.2 were considered for the multivariable analysis. A p-value less than or equal to 0.05 was considered statistically significant. The presence of interaction was assessed by using the log-likelihood test comparing the full and reduced models. Confounding variables were assessed by comparing prevalence ratios of unadjusted and adjusted models and those that caused a difference of at least 10% were considered confounders. We however did not find any significant interaction or confounding. The goodness of fit of the regression model to the data was assessed using the deviance test.

-The reference category for first ANC has been changed to “first trimester of ANC” as shown in table 3

**Reviewer** #1

**General Comments**: This manuscript “Optimal utilization of Prevention of Mother –to Child Transmission of HIV services among adolescents under group versus focused antenatal care in Eastern Uganda” addresses a timely and challenging topic—how best to ensure uptake of PMTCT services by adolescents. The cross sectional study conducted in postpartum and immunization clinics, reports encouraging results with use of age and gestational grouping of antenatal services (“G-ANC) in terms of optimal utilization of PMTCT services by adolescents. “ As their outcome of interest the authors chose being up to date with HIV counseling and testing during the antenatal period among 528 pregnant adolescents; and assessed clinical and demographic factors associated with this outcome. They reported that 75% of the adolescents who received Group focused antenatal care received test results compared to 42% of those receiving standard focused care. Factors associated with adolescent receipt of antenatal HCT included participation in Group ANC services, partner being tested for HIV, facility level, and earlier presentation for prenatal services.
While the findings are of interest, the manuscript had a number of concerns which detract from confidence in the findings. This included a number of proofing errors in the abstract and main manuscript with discrepancies between text results and the bivariate Table results; and some instances where reported prevalence ratios do not fall within the listed confidence intervals. In addition, the cascade of PMTCT services as described includes multiple other PMTCT outcomes of interest besides receipt of HCT services during the antenatal period, which were apparently were not assessed as part of the study analyses. If other parts of the PMTCT cascade were actually included in a composite outcome, this needs to be described/clarified in the methods text. And there is concern that the group of adolescents described are a very select group in terms of their high rates of health seeking behavior which makes generalizability of the results questionable.

**Response**

Thank you for this summary of the paper. Our responses to each of the individual comments are provided below.

According to the World Health Organization, the PMTCT strategy is a cascade of events that enable the identification of an HIV-infected pregnant woman and the prevention of HIV transmission to her infant. It comprises: attending antenatal care (ANC), HIV counseling and testing (HCT), and receiving HIV test results. If HIV negative, subsequent retesting in the third trimester, shortly after delivery, or within three months. If positive, antiretroviral therapy (ARVs) for the mother under option B plus (1), Nevirapine syrup (NVP), cotrimoxazole prophylaxis, and ARVs for the infant. Safe delivery, safe infant feeding, early infant diagnosis within 6 weeks, DNA PCR done 6 weeks after ceasing breastfeeding, serology at 18 months if DNA PCR is negative, and finally linkage into care and treatment if infant turns out positive (1) (2).

We therefore for HIV negative mothers considered first HIV counseling and testing before the third trimester, receipt of results and subsequent re-testing as recommended. For HIV positive mothers however, we looked at whether they were initiated on ART under option B plus. In addition, for the infants we asked if: there was safe infant delivery (delivery in a health facility), safe infant feeding (exclusive breast feeding for the first 6 months), infant testing, and receipt of results, nevirapine was given for six weeks, and cotrimoxazole was given until status was confirmed. Infant testing entailed early infant diagnosis (DNA PCR within 6 weeks), if the test turned positive, ART was initiated and the infant linked into care. We did not assess for the second DNA PCR test or serology as the infants had to be 9 months of age or less.

**Comment #1 (Abstract)**

The numbers in the abstract results for independent factors associated with optimal PMTCT do not exactly match those in Table 2 –e.g. partner tested results. In addition, there appear to be typing errors where the PR does not fall within the CI range—partner tested as an example. These need to be harmonized

**Response**

The results in the abstract have been harmonized to reflect those from the multivariable analysis reflected in table 3

**Comment #2 (Introduction/Background)**

Generally well written with good description of the PMTCT cascade. Sentence Line 59-60 is lacking several verbs and needs to be rewritten.

**Response**

This manuscript has been edited and the spelling errors are taken care of.

**Comment #3 (Study participants and sampling)**

The inclusion criteria as stated required that the adolescent mothers had received at least 18 months of care if HIV+ and 9 months if HIV negative, (p 5, line 100) and for the infants to be 9 months or less of age; so, this is a very select group in terms of HIV infected adolescent mothers being already engaged in HIV health care. It also implies that HIV+ adolescent mothers would have known their status prior to the pregnancy if they had to have received 18 months of HIV care while their infants had to be aged 9 months or less, which again would be a very select group of adolescents who would have been aware of their HIV status prior to testing in ANC.

**Response**

We recognize that this is a major limitation of this study as this group of older adolescents most likely have good health-seeking behavior and this affects the generalizability of the findings. We however believe that these findings can be generalized to older adolescents seeking health care at other health facilities.

**Comment #4 (Data analysis)**
Statistical methods appear appropriate and sample size adequate to address the stated antenatal HCT outcome of interest.

**Response**

We have re-analyzed the data using fixed effects regression modeling as advised by academic editor review.

**Comment #5 (Table 1 and text)**
It would be helpful to state the full range of age for the adolescents at the time of their enrolment in the study; as the vast majority >80%) were at least 18 years of age. Were most of them in their early 20’s or is upper age range just to age 19 years, and what is the youngest age of the pregnant adolescents included in the analyses?

**Response**

Table 1 has been edited to include the median age (18 years) and the 25^th^ and 75^th^ percentiles. The youngest adolescent was 14 years while the oldest was 19 years old- lines 212 to 213.

**Comment #6 (Table 2):**

Table 2 which presents the bivariate results needed a number of corrective edits including:
• While the text, line 176, p 11, for the bivariate analyses presents PR and CI for the G-ANC and F-ANC groups, this G-ANC and F-ANC data are not presented in Table 2.
• Re the age , column Yes, the percent >= 18 years is missing
• Re variable first ANC visit for first trimester there is an extra 1 typo in the confidence intervals.
• HIV test results there is a missing upper confidence limit
• In addition the text page lines 1, lines 177 -178 stated numbers for the PR and CI do not match the Table 2 numbers with regards to : 1st ANC visit in first or second trimester, or regarding partner tested, or for HCIV clinic.

**Response**

- The bivariate analysis by mode of ANC has been added to table 3, factors associated with optimal utilization of PMTCT of HIV services
- The proportion for age category ≥18 years has been added in table 3
- The confidence intervals for “first ANC” have been edited in table 3
- upper confidence limit has been added to HIV test results in table 3
- The numbers in the text and table 3 have been harmonized for trimester of first ANC visit, partner testing status.

**Comment #7 (**Discussion**)**:

Generally well written. Of interest is the substantially higher uptake of PTMCT HCT for both the G-ANC and F-ANC among adolescents when compared to central Uganda. The authors should comment on what regional differences may account for this finding. Also of interest and surprising is the relatively high proportion of adolescents who made their first ANC visit in the first trimester for either the G-ANC (41.7%) or the F-ANC (42.1%). The authors could comment on how this early health seeking behavior among the adolescents was achieved!

In addition to the limitations stated, it would also be important in terms of potential generalizability of findings that this was a select group of older adolescents with high health seeking behavior and therefore may not be applicable to younger pregnant adolescents in resource limited international settings.

**Response**

The higher optimal utilization of PMTCT of HIV services in the eastern region compared to the central observed could be because of the intensified efforts to promote maternal, child and adolescent health in the Busoga region by both the Ministry of Health and implementing partners. These include the Regional health integration to enhance services in the Busoga region (3), the government of Korea’s partnership with WHO and the Ministry of Health to support maternal, adolescent, and child health delivery in Busoga(4) among others (5, 6). As such, there has been collaboration with village health teams for mapping of pregnant adolescents; who are then linked to health care. This could also explain the high numbers of adolescent mothers attending ANC early i.e. in the first trimester. The observed difference could also be because the findings in central Uganda were from a study carried out in a national referral hospital, Mulago National Referral Hospital. There was likely difficulty in follow-up and as such poorer PMTCT service utilization.

We recognize that the generalizability of our findings is a major limitation of this study as this group of adolescents was older and also most likely have good health-seeking behavior. We however believe that these findings can be generalized to older adolescents seeking health care at health facilities. This has been adjusted in lines 355 to 357

.

1.	Ministry Of Health. Consolidated guidelines for the prevention and treatment of HIV and AIDS in Uganda. 2018.

2.	Hamilton E, Bossiky B, Ditekemena J, Esiru G, Fwamba F, Goga AE, et al. Using the PMTCT Cascade to Accelerate Achievement of the Global Plan Goals. J Acquir Immune Defic Syndr. 2017;75 Suppl 1(1):S27-S35.

3.	University Research Co. Regional Health Integration to Enhance Services in East Central Uganda Activity (2016-2022). 2016.

4.	World Health Organisation. The Government of South Korea and WHO extend support worth Ten Million Dollars towards Health System Strengthening for Improving Reproductive, Maternal, Child, and Adolescent Health-Service Delivery in Five districts in Busoga sub-region in Uganda. 2020.

5.	Reach a hand. Get Up Speak Out for youth rights. 2020.

6.	Ministry of Health. THE NATIONAL STRATEGY TO END CHILD MARRIAGE AND TEENAGE PREGNANCY 2020.
