# Supplementary material for: Optimal utilization of prevention of mother-to-child transmission of HIV services among adolescents under group versus focused antenatal care in Eastern Uganda
Source: PLoS One. 2022 Nov 1;17(11):e0275905. doi: 10.1371/journal.pone.0275905 (PMC9624396; doi:10.1371/journal.pone.0275905)
Supplement: Attachment — Submitted filename: Response to Reviewers.docx [file pone.0275905.s003.docx]

August 11, 2022

To: Orvalho Augusto

Academic Editor

PLOS ONE

Reference number: PONE-D-21-31253

Thank you for your positive consideration and for inviting us to submit the revised version of our manuscript entitled: “*Optimal utilization of Prevention of Mother-to-Child Transmission of HIV services among adolescents under Group versus Focused antenatal care in Eastern Uganda*.” The responses to each of the reviewers' comments are listed below.

**Responses to the reviewers’ comments**

**Academic editor**

**General comment**

Please review your reference list to ensure that it is complete and correct. If you have cited papers that have been retracted, please include the rationale for doing so in the manuscript text, or remove these references and replace them with relevant current references. Any changes to the reference list should be mentioned in the rebuttal letter that accompanies your revised manuscript. If you need to cite a retracted article, indicate the article’s retracted status in the References list and also include a citation and full reference for the retraction notice.

**Response**:

The reference list has been revised. References: 2,3,7,8, and 9 were previously retracted and have been updated with new literature. References: 4,6,8,15,17, 27,28, 29, 30, 31, 32, 33 were edited as they were incomplete. References 39-40 were replaced with more relevant references

Reviewer #2. The authors have responded satisfactorily to the previous reviewers' comments. My comments are mostly editorial.

**Comment 1**: Line 78. For clarity it would be helpful to define "safe delivery" and "safe infant feeding".

**Response**: Safe delivery, defined as delivery in a health facility by a qualified health worker has been added to line 78/79. Safe infant feeding defined as exclusive breastfeeding for six months, complementary feeding for up to 12 months for HIV-exposed infant, and 24 months for HIV-positive infants has been added to line 79/80.

**Comment 2**. Line 93, place comma after "Although G-ANC",

**Response**. A comma has been added as advised (line 95).

**Comment 3**. Line 103. Better to say "This was a cross-sectional study..."

**Response**. This has been edited as suggested (line 105).

**Comment 4**. Line 105. Define HCIV. Many readers are not familiar with the Uganda health care system.

**Response**. The definition of HCIV, a mini-hospital headed by a senior medical officer; has been added (line 107-108)

**Comment 5**. Line 142. Again define "safe delivery", "safe infant feeding".

**Response**. The definitions for safe delivery and safe infant feeding have been added on lines 78/79 and 79/80 respectively

**Comment 6**. Line 144. DNA PCR within 6 weeks. Is the process not DNA PCR within 6-8 weeks? Within 6 weeks implies this assessment being even within 2 weeks, which is incorrect.

Response. Thank you for this clarification. Yes, the first DNA PCR should be within 6-8 weeks. This has been edited on line 147

**Comment 7**. Line 164. Need to describe the interviewers, their qualifications and training.

**Response**. We trained 6 interviewers (diploma-level nurses) for 4 days on the questionnaire, data collection procedures, and sampling methods. This has been added on line 162

**Comment 8**. Line 166. A better term than "cross-examined" might be "cross-referenced", "correlated with", "checked with", "compared with".

**Response**. The term has been changed to cross-referenced (line 170)

**Comment 9**. Line 263/264. Need to provide evidence/reference for the statement that "...G-ANC was attributed to the participatory nature of G-ANC resulting in a better relationship between health care workers."

**Response**. We think that the observed better optimal utilization of PMTCT of HIV services under G-ANC could be attributed to improved client satisfaction and prenatal service utilization. The improved client satisfaction has been attributed to the participatory nature of G-ANC which improves relationships between health care workers and mothers as well as among the mothers (Line 267-269). References to support these statements have been added to line 270.

**Comment 10**. Line 292. "Larger catchment areas and thus populations in health facilities result in difficulty in follow-up." This statement is not clear. If the authors are trying to say that larger catchment areas with larger populations make follow-up more difficult, they need to provide evidence.

**Response**.

The statement, line 297/298, has been edited to ‘Larger catchment areas with larger populations likely result in high workloads which affects the quality of patient care’. References to support this statement have been added to line 298/299
